# Forever Love: The Hitherto Earliest Record of Copulating Insects from the Middle Jurassic of China

**DOI:** 10.1371/journal.pone.0078188

**Published:** 2013-11-06

**Authors:** Shu Li, Chungkun Shih, Chen Wang, Hong Pang, Dong Ren

**Affiliations:** 1 Key Laboratory of Insect Evolution & Environmental Changes, Capital Normal University, Beijing, P.R. China; 2 Institute of Plant and Environmental Protection, Beijing Academy of Agriculture and Forestry Sciences, Beijing, China; 3 State Key Laboratory of Biocontrol and Institute of Entomology, Sun Yat-sen University, Guangzhou, P.R. China; Universidade de São Paulo, Faculdade de Filosofia Ciências e Letras de Ribeirão Preto, Brazil

## Abstract

**Background:**

Mating behaviors have been widely studied for extant insects. However, cases of mating individuals are particularly rare in the fossil record of insects, and most of them involved preservation in amber while only in rare cases found in compression fossils. This considerably limits our knowledge of mating position and genitalia orientation during the Mesozoic, and hinders our understanding of the evolution of mating behaviors in this major component of modern ecosystems.

**Principal Finding:**

Here we report a pair of copulating froghoppers, *Anthoscytina perpetua* sp. nov., referable to the Procercopidae, from the Middle Jurassic of northeastern China. They exhibit belly-to-belly mating position as preserved, with male's aedeagus inserting into the female's bursa copulatrix. Abdominal segments 8 to 9 of male are disarticulated suggesting these segments were twisted and flexed during mating. Due to potential taphonomic effect, we cannot rule out that they might have taken side-by-side position, as in extant froghoppers. Genitalia of male and female, based on paratypes, show symmetric structures.

**Conclusions/Significance:**

Our findings, consistent with those of extant froghoppers, indicate froghoppers' genitalic symmetry and mating position have remained static for over 165 million years.

## Introduction

Mating behaviors for extant insects have been studied and documented, for example, for froghoppers[1], scorpionflies[2] and planthoppers[3]. However, fossil records of unequivocal insect mating are fairly sparse. Boucot and Poinar[4] listed 33 instances of fossilized mating insects, such as fireflies, mosquitoes, planthoppers, leafhoppers, water striders, bees and ants, 27 of which are preserved in amber, others on compression fossils. The hitherto oldest example of copulation in fossil insects is a pair of chironomids (Diptera) discovered in Early Cretaceous amber from Lebanon [4], [5].

Herein, we report a pair of well-preserved copulating froghoppers, *Anthoscytina perpetua* sp. nov., referable to the Procercopidae, from Jiulongshan Formation at the Daohugou Village in northeastern China. Procercopidae is an extinct family in the superfamily of froghoppers, Cercopoidea Leach, 1815. Froghoppers get their name because the adults hop around on plants and shrubs like tiny frogs. The nymphs of froghoppers are called spittlebugs because they cover themselves with foaming spittle, composed of tiny air bubbles trapped in secretions from their Malpighian tubules, which provides protection from predation, parasitism and desiccation[6]. This discovery of the earliest record of copulating insects hitherto sheds light on the evolution of mating behavior in this group of insects.

Daohugou Village (N41°18′38″, E119°13′20″) is located in Shantou Township, Ningcheng County, Inner Mongolia Autonomous Region, northeastern China. The Jiulongshan Formation is considered to be of late Middle Jurassic age (Bathonian–Callovian boundary interval, 164–165 Ma), based on Ar-Ar and SHRIMP U-Pb dating results [7]–[10]. Currently, about 19 insect orders have been reported from this locality[11].

## Materials and Methods

### Materials

We examined more than 1200 specimens from the locality of Daohugou, Inner Mongolia, China. All fossil materials studied are housed in the fossil insect collection of the Key Laboratory of Insect Evolution and Environmental Changes, College of Life Sciences, Capital Normal University, Beijing, China (CNUB; Dong Ren, Curator). The extant *Cosmoscarta heros* (Fabricius) specimens are from the Insect Collection of Sun Yat-sen University, Guangzhou, China.

### Methods

The specimens were examined by a LEICA MZ12.5 dissecting microscope and illustrated with the aid of a drawing tube attachment. Fossil photographs were taken by Nikon Digital Camera DXM1200C.

We follow the traditional terminologies of Cercopoidea[12] and Nel et al.[13]. Venation abbreviations used in the text and Figures: Cu, Cubitus; CuA, Cubitus Anterior; CuP, Cubitus Posterior; M, Media; MA, Media Anterior; MP, Media Posterior; Pcu, Postcubitus; R, Radius; RA, Radius Anterior; RP, Radius Posterior; ScA, Subcosta Anterior; ScP, Subcosta Posterior; m-cua, veinlet between MP and CuA, r-m; veinlet between R and M; ir, veinlet between RA and RP.

### Nomenclatural Acts

The electronic edition of this article conforms to the requirements of the amended International Code of Zoological Nomenclature, and hence the new names contained herein are available under the Code from the electronic edition of this article. This published work and the nomenclatural acts it contains have been registered in Zoobank, the online registration system for the ICZN. The Zoobank LSIDs (Life Science Identifiers) can be resolved and the associated information viewed through any standard web browser by appending the LSID to the prefix “Http://zoobank.org”. The ISID for this publication is: urn: lsid: zoobank.org: pub: AF664AE6-A000-45B9-B9BC-6759CD0F63EE. The electronic edition of this work was published in a journal with an ISSN, and has been archived and is available from the following digital repositories: PubMed Central and LOCKSS.

## Results

### Systematic Palaeontology

Order Hemiptera Linnaeus, 1758

Suborder Cicadomorpha Evans, 1946

Superfamily Cercopoidea Leach, 1815

Family Procercopidae Handlirsch, 1906

Genus *Anthoscytina* Hong, 1983

#### Type species

*Anthoscytina longa* Hong, 1983[14] (Middle Jurassic of Haifanggou, Beipiao City, Liaoning, China).

#### Other included species

*A. reducta* (Becker-Migdisova, 1949)[15] (Lower Jurassic of Kyzyl-Kiya, Kyrgyzstan); *A. daica* Shcherbakov, 1988[16] (Upper Jurassic–Lower Cretaceous, Glushkovo Formation of Chita, Siberia, Russia); *A. parallelica* Ren, Lu, et Guo, 1995[17] (Middle Jurassic of Zhouyingzi, Hebei, China); and *A. aphthosa* Ren, Yin, et Dou, 1998[18] (Lower Cretaceous, Yixian Formation of Beipiao, China); and *A. perpetua* Li, Shih et Ren, sp. nov.

### *Anthoscytina perpetua* Li, Shih et Ren, sp. nov. (Figs. 1-4)

urn:lsid:zoobank.org:act:7DDCC368-3CBA-4815-9DA1-762AC77DE6DE

#### Diagnosis

Forewing slender, RA simple (vs. 2-4 branches in *A. reducta*, *A. daica* and *A. parallelica*); M branching into MA and MP at distal fifth of wing (vs. at 3/4 of wing in *A. longa*); CuA_1_ twice as long as CuA_2_ (vs. 1.5 times as long as CuA_2_ in *A. longa*); crossvein ir at level of crossvein r-m, apical of the crossvein m-cua (vs. ir directly distal to other veinlets in *A. aphthosa*). Hind wing with crossvein r-m between MA and RP, slightly distal to bifurcation of M.

#### Description

**Figure 1:**
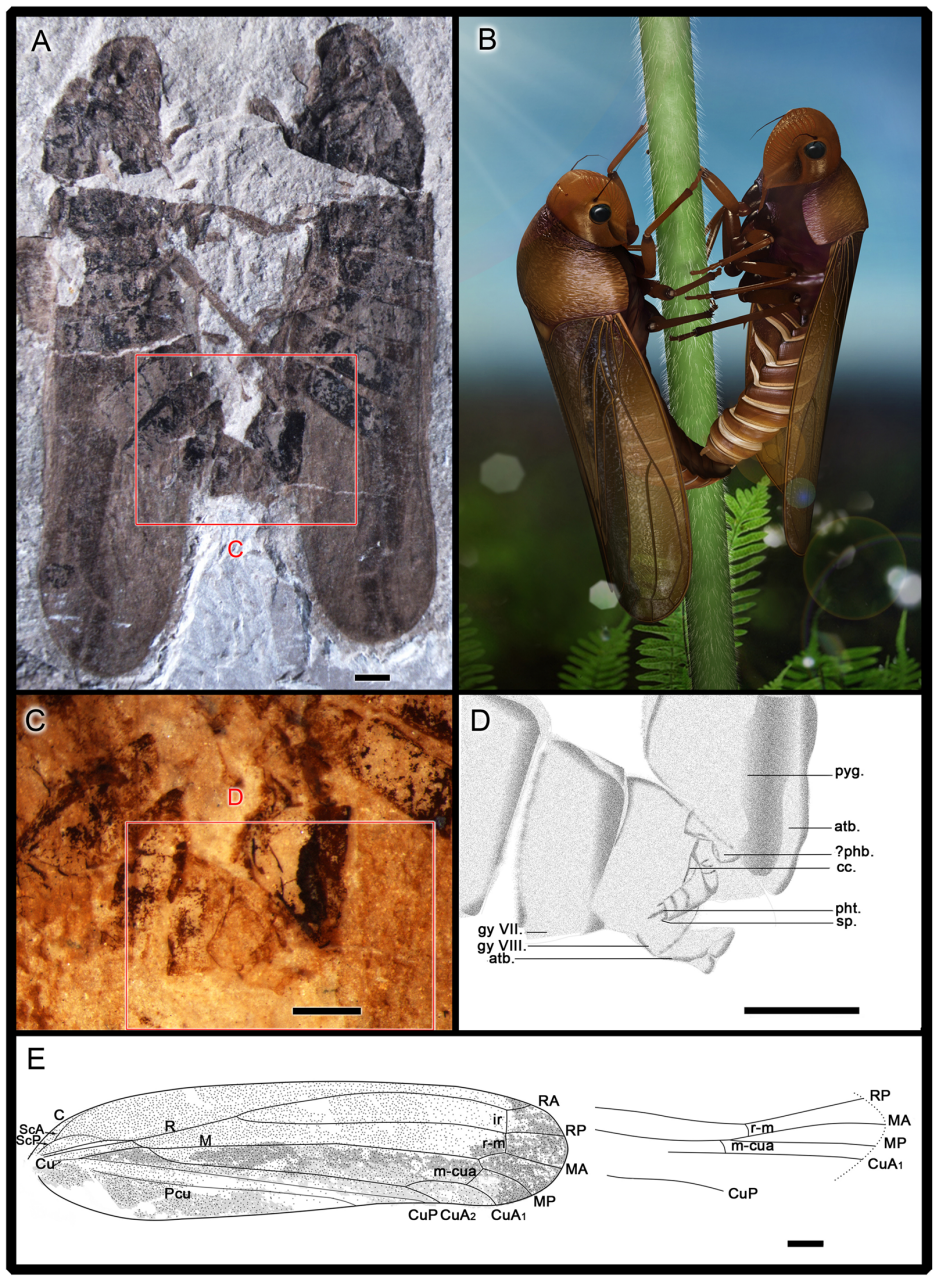
*Anthoscytina perpetua* Li, Shih et Ren, sp. nov. A–D, holotype. male, on the right (CNU-HEM-NN2012002 p) and allotype. female, on the left (CNU-HEM-NN2012003 p). **A**, photograph of habitus. **B**, 3-D ecological reconstruction. **C**, photograph of male and female genitalia in copulation, under alcohol. **D**, interpretative drawing of C. **E**, paratype. CNU-HEM-NN2010003, interpretative drawings of venations of the forewing and hind wing. pyg., pygofer; atb., anal tube; phb., phallobase; cc., corpus connective; pht., phallotrema; sp., sclerotized process; gy., gonapophyses. Scale bars  = 1 mm (**A, C, D, E**).

**Figure 2:**
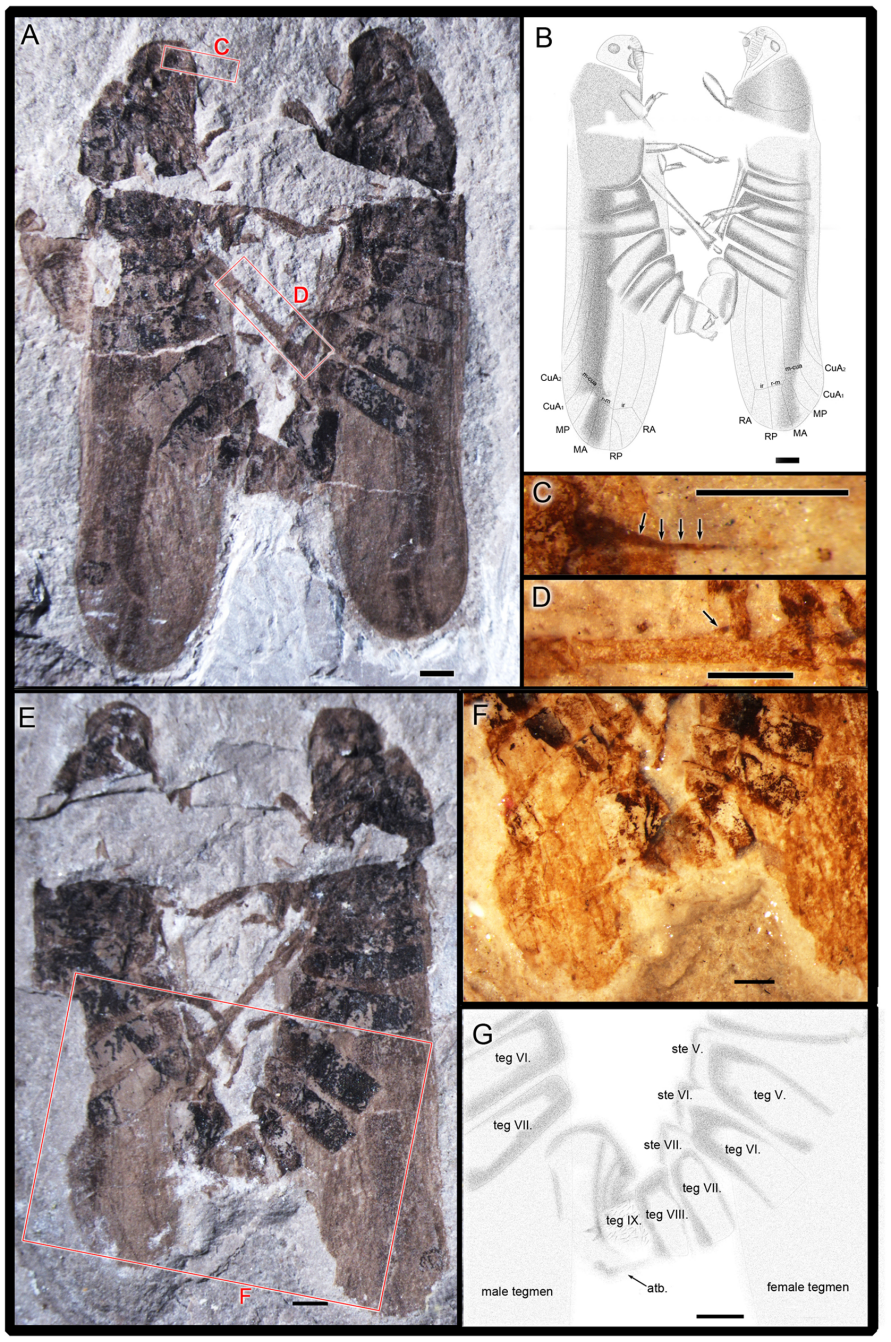
*Anthoscytina perpetua* Li, Shih et Ren, sp. nov. A–B, holotype. (CNU-HEM-NN2012002 p, male, on the right) and allotype (CNU-HEM-NN2012003 p, female, on the left). **A**, photograph of habitus. **B**, interpretative drawing. **C–D**, paratype. CNU-HEM-NN2010003 p. **C**, photograph of antenna, under alcohol. **D**, photograph of hindleg, under alcohol. **E–F**, holotype. (CNU-HEM-NN2012002 c, male, on the left) and allotype (CNU-HEM-NN2012003 c, female, on the right). **E**, photograph of habitus. **F**, photograph of male and female genitalia in copulation, under alcohol. **G**, interpretative drawing of male and female genitalia in copulation. teg., tergite; ste., sternite; atb., anal tube. Scale bars  = 1 mm for all.

**Figure 3:**
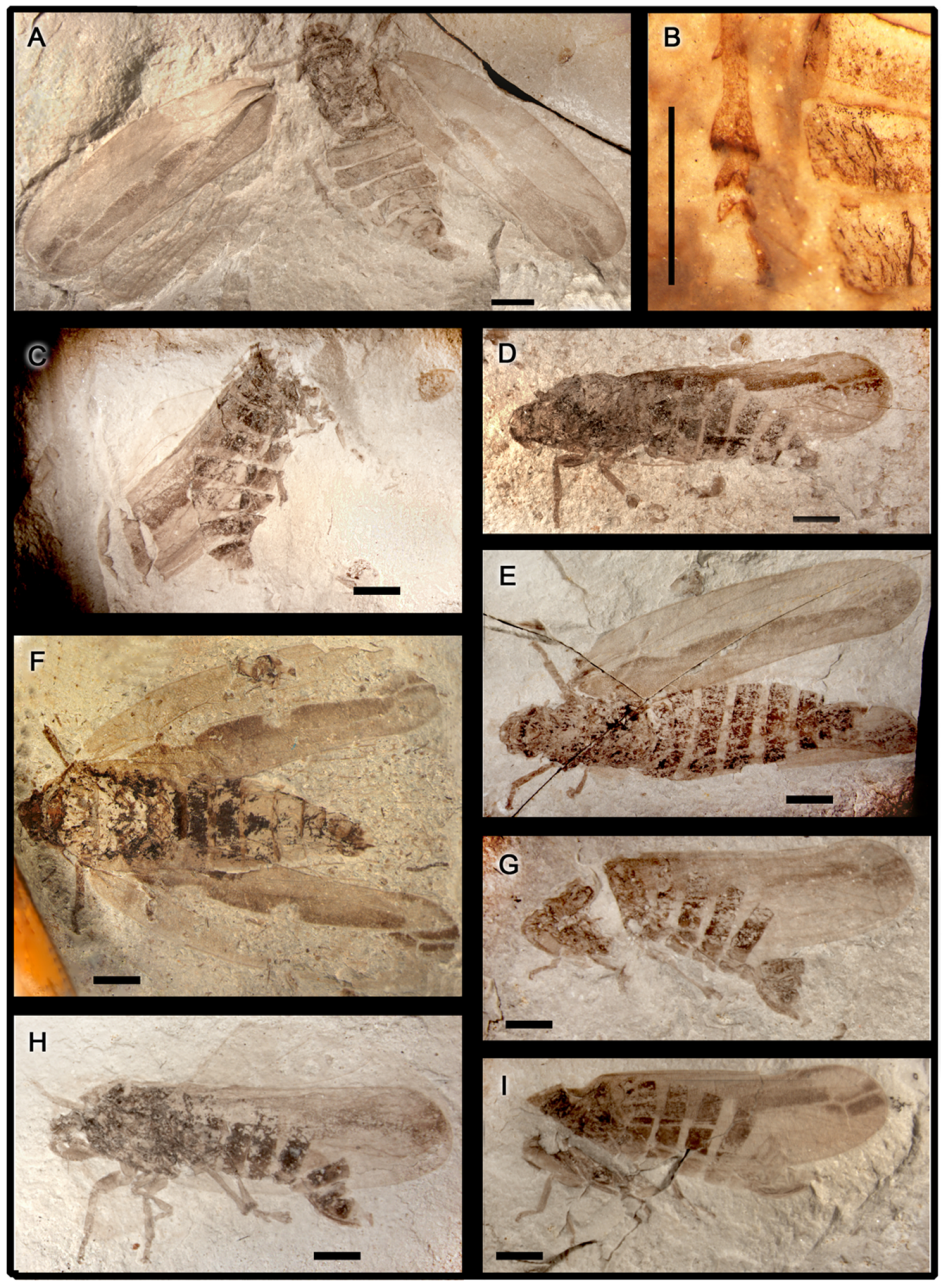
*Anthoscytina perpetua* Li, Shih et Ren, sp. nov. **A–I**, photographs of paratype specimens: **A**, CNU-HEM-NN2010014, male. **B**, hind leg with a lateral spine, under alcohol, CNU-HEM-NN2010014. **C**, CNU-HEM-NN2010247, male. **D**, CNU-HEM-NN2010236, male. **E**, CNU-HEM-NN2010003, female. **F**, CNU-HEM-NN2010006, female. **G**, CNU-HEM-NN2010035p, female. **H**, CNU-HEM-NN2010046p, female. **I**, CNU-HEM-NN2010042, female. Scale bars  = 2 mm for all.

Body 15-17 mm long including forewings in repose (Figs. 1A, 2A, E, 3); head narrower than pronotum, with declivous inflated clypeus; eyes ovoid, antenna with 4 segments visible (Fig. 2C); postclypeus swollen, about 0.9 mm long, and 0.3 mm wide, with distinct transverse grooves; rostrum very long, well extending beyond middle coxae, about 2.4 mm long. Pronotum greatly expanded, 2 times as long as vertex at mid length; mesonotum and scutellum about 1.43 mm long. Hind tibia with 1 lateral spine (Figs. 2D, 3B).

Forewing elongate, length 13.2–15.3 mm, width 3.5–4.3 mm, length/width ratio 3.2–4.3, based on holotype, allotype (Fig. 2B) and paratypes listed in the referred material (Fig. 3). ScA terminating at running to costal margin, ScP arched and fusing with R at basal 1/7 of wing length. R branching into RA and RP at basal 2/5 of wing length. RA simple, a sinuous crossvein ir between RA and RP at basal 8/9 of wing length. RP with 1–2 branches at basal 9/10 of wing length. Crossvein r-m meeting RP at the same point of crossvein ir. M branching into MA and MP at basal 5/6 of wing length. A crossvein m-cua between M and CuA_1_. Crossvein ir at level of crossvein r-m, apical of crossvein m-cua. Stem Cu bifurcating into CuA and CuP at wing base. CuA curving anteriorly and meeting M, then bending posteriorly, CuA branching into CuA_1_ and CuA_2_ at basal 2/3 of wing length. Middle part of CuA_1_ curving anteriorly. CuA_1_ twice as long as CuA_2_. CuP straight, reaching posterior margin at the same point as CuA_2_, about 3/4 of wing length, forming a long clavus. Vein Pcu slightly curved, ending at about midpoint of wing. Fore wing membrane with granules. Posterior part of membrane infuscate.

Hind wing partly preserved in paratype CNU-HEM-NN2010003 (Figs. 1E, 3E). Vein M with 2 branches, crossvein r-m between MA and RP, slightly distal to bifurcation of M. CuA_1_ connecting with M by a crossvein m-cua basal to bifurcation of M.

**Figure 4:**
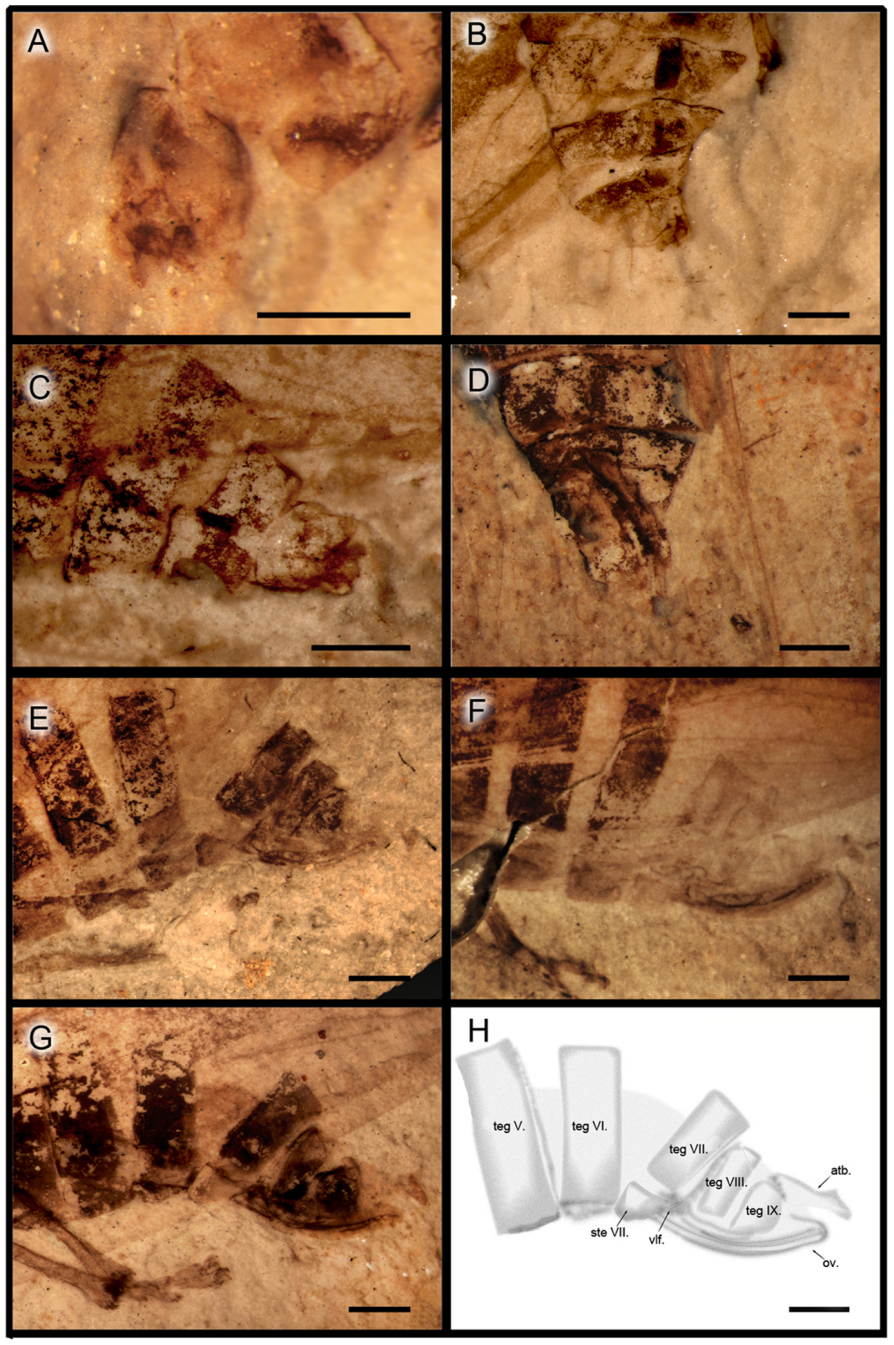
*Anthoscytina perpetua* Li, Shih et Ren, sp. nov. A-G photographs of paratype specimens: **A**, CNU-HEM-NN2010014, male genitalia, dorsal view. **B**, CNU-HEM-NN2010247c male genitalia, ventrolateral view. **C**, CNU-HEM-NN2010236, male genitalia, dorsolateral view. **D**, CNU-HEM-NN2010006p, female genitalia, dorsal view. **E**, CNU-HEM-NN2010035p, female genitalia, lateral view. **F**, CNU-HEM-NN2010042, female genitalia, lateral view. **G**, CNU-HEM-NN2010046p, female genitalia, lateral view. **H**, CNU-HEM-NN2010046p, interpretative drawing of female genitalia in G. teg., tergite; atb., anal tube; ste., sternite; vlf., valvifer; ov., valvula of ovipositor. Scale bars  = 1 mm for all.

Male genitalia: Pygofer symmetrical in lateral view (clearly preserved), approximately 2 times as long as broad, posterior margin gently produced caudad in lateral view; accommodating anal tube at the apex (Figs. 1C, D, 4A–C,). Segments 8 to 9 disarticulated from segment 7. Aedeagus elongate, tubular, when copulating inserted inside the bursa copulatrix of the female, shaft of aedeagus with external sclerotization from base to turning point, with one apical spine and phallotrema pin-like (Figs. 1C, D). Gonostyli of paratypes CNU-HEM-NN2010014 and CNU-HEM-NN2010236 symmetrical, short and broad, fused with pygofers at base (Figs. 3A, D, 4A, C).

Female genitalia: Anal tube elongate, apical margin concave in lateral view. Valvulae not visible in copulating female (Figs. 1A, C, D, 2E–G). In the paratypes of female specimens (Figs. 4D–H), valvulae symmetrical almost rectangular, sclerotized plates in lateral view, but in dorsal view with some distinct hairs on the cauda (IX tergite) (Figs. 2F, G).

#### Etymology

From the Latin *perpet*, eternal love, in reference to this everlasting copulation.

#### Holotype

CNU-HEM-NN2012002 p/c (male) (Figs. 1, 2)

#### Referred material

Allotype: CNU-HEM-NN2012003p/c (female) (Figs. 1, 2); paratypes: males, CNU-HEM-NN2010014, CNU-HEM-NN201049, CNU-HEM-NN2010236, CNU-HEM-NN2010247; females, CNU-HEM-NN2010003, CNU-HEM-NN2010006, CNU-HEM-NN2010010, CNU-HEM-NN2010035 p/c, CNU-HEM-NN2010042, CNU-HEM-NN2010046, CNU-HEM-NN2010078, CNU-HEM-NN2010221, CNU-HEM-NN2010243. (Figs. 3, 4)

#### Locality and age

Daohugou Village, Ningcheng County, Chifeng City, Inner Mongolia, China. Jiulongshan Formation, late Middle Jurassic, the Callovian–Bathonian boundary.

## Discussion

**Figure 5:**
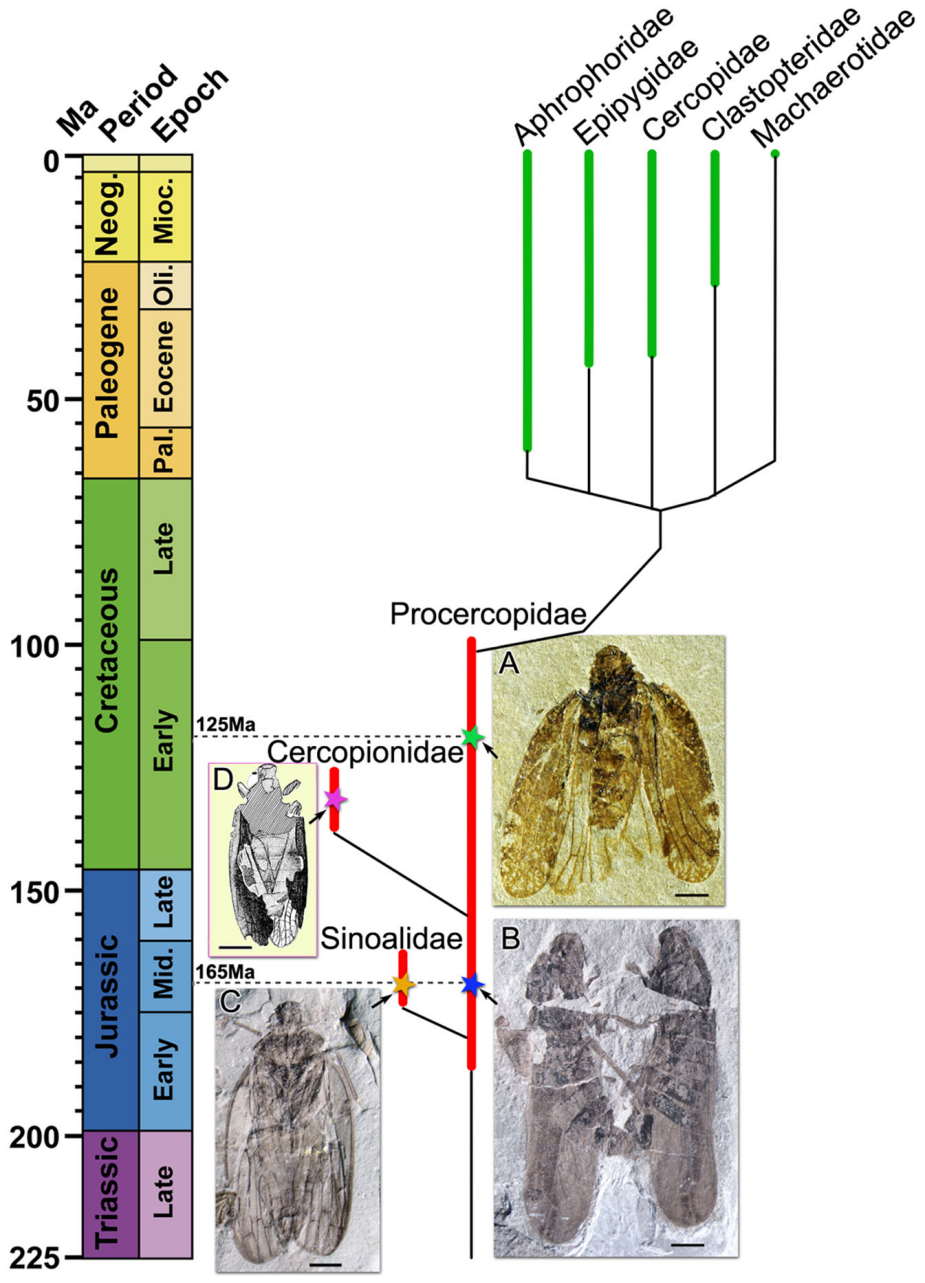
Tentative reconstruction of phylogenetic relationships within the Cercopoidea. Morphological data in extant and fossil taxa have been considered, as well as phylogenetic scheme of the Clypeata proposed by Wang et al. [19]. **A**, *Anthoscytina aphthosa* Ren, Yin and Dou, 1998 from the Early Cretaceous of Yixian Formation, China. **B**, *Anthoscytina perpetua* Li, Shih et Ren, sp. nov. from the Middle Jurassic of Jiulongshan Formation, China. **C**, *Jiania gracila* Wang et Szwedo, 2012 from the Middle Jurassic of Jiulongshan Formation,China. **D**, *Cercopion reticulata* Hamilton, 1990 from the Early Cretaceous of Santana Formation, Brazil. Scale bars  = 2 mm for all.

The superfamily of froghoppers Cercopoidea Leach, 1815 comprises approximately 3000 described species distributed in five extant families. Following Wang et al. [19], a tentative reconstruction of the phylogenetic relationships within the Cercopoidea is proposed in Fig. 5. Although fossil records of Cercopoidea are lacking during the Late Cretaceous, Wang et al. have hypothesized that extant families, with the earliest fossil records from Paleocene, evolved from Procercopidae[19].

On the mating pair, the male's aedeagus is inserted inside the bursa copulatrix of the female. The shaft of aedeagus has external sclerotization from base to turning point, with an apical spine and a pin-like phallotrema (Figs. 1C, D). Abdominal segments 8 and 9 of the male are disarticulated (Figs. 1A, C, 2E–G), suggesting that these segments might have been twisted and flexed during mating. The female's valvulae are not visible on the fossils, but anal tube is distinctly elongate and apical margin concave. As shown by representative paratypes in Figs. 4A–G, male and female genitalia are symmetrical. The copulating pair exhibit belly-to-belly mating position as preserved. However, due to the potential taphonomic effect, we cannot rule out that they might have taken a side-by-side position when alive, as do extant froghopper taxa[1], [6], [20].

Mating positions and the evolution of asymmetric insect genitalia have been reviewed by Huber et al[21] and further reported by Huber[22]. Huber et al. proposed that the female-above mating position is plesiomorphic for Neoptera. The next process involves a change of positions to male-above, belly-to-belly, or side-by-side, in which the male's genitalia actually contact the female from below as in a female-above position. These positions presumably allow a better control for the males during mating. In most of these positions, both females and males have symmetric genitalia, except for the cases that the side of approach becomes fixed (“one-sided position”), genitalia become asymmetric. For end-to-end and belly-to-belly positions, insects usually rotate the abdomen or the genitalia by 180 degree[21]. Genital asymmetry is a recurring phenomenon in insect morphology and current data suggest that it has arisen multiple times independently in several neopteran orders[22].

**Figure 6:**
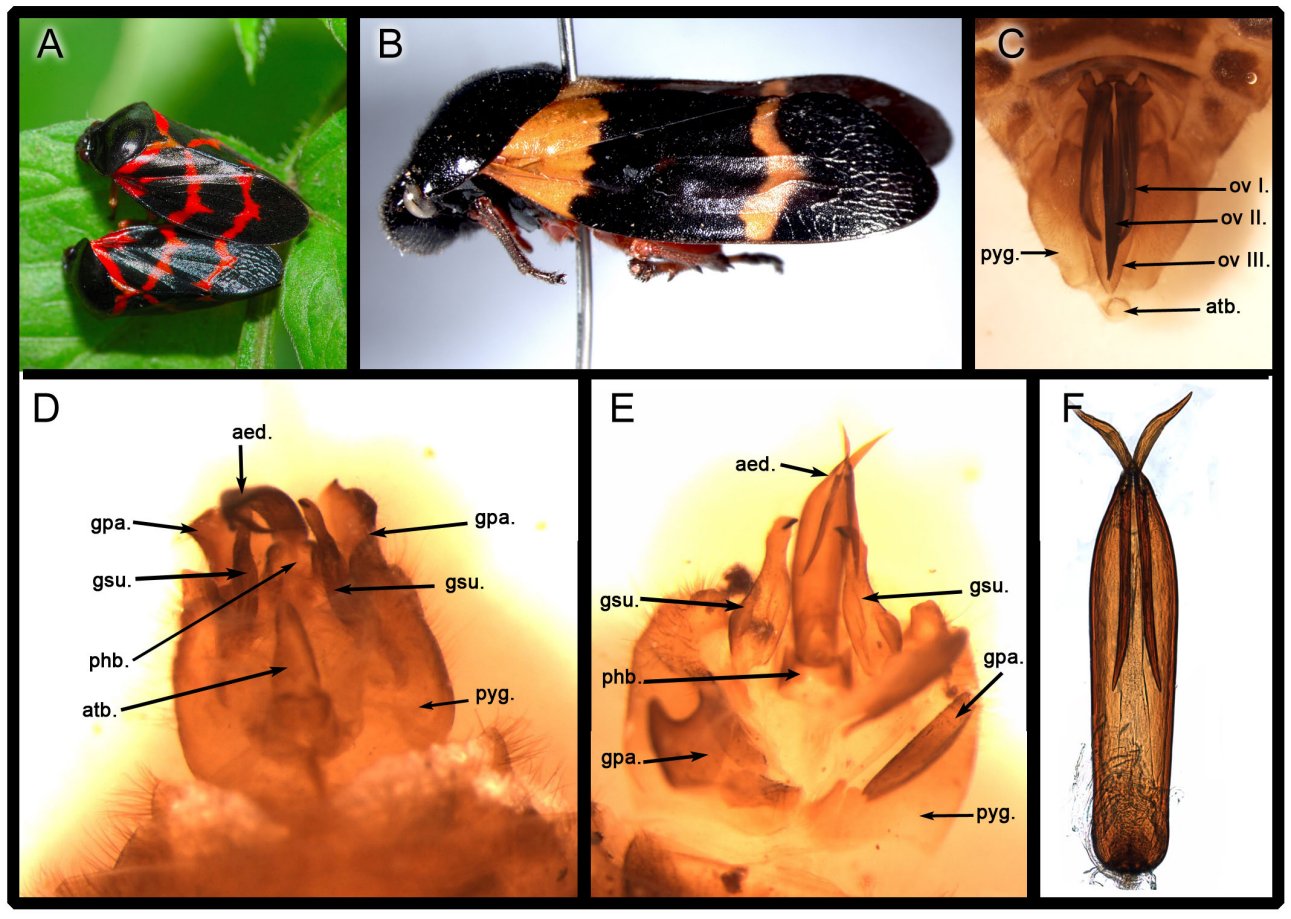
Mating position and genitalia of representative extant froghoppers. **A**, froghoppers in copulation, photo credit: Jason Shih. **B–F**, *Cosmoscarta heros* (Fabricius). **B**, lateral habitus. **C**, ovipositor ventral view. **D**, male genitalia, dorsal view. **E**, male genitalia, ventral view. **F**, aedeagus, dorsal view. pyg., pygofer; ov., valvula of ovipositor; atb., anal tube; aed., aedeagus; gsu., gonstylus; gpa., genital plates; phb., phallobase.

Extant froghoppers, both males and females, have symmetric genitalia [21], [23]–[25]. The genitalia of a representative extant froghopper of *Cosmoscarta heros* (Fabricius) are shown in Figs. 6B–F. The male aedeagus curves upward dorsally (Figs. 6D, E), indicating that the male rotates and flexes its terminalia during side-by-side (or belly-to-belly) mating. In the CNU's extensive collection of more than 200 thousand insect fossils, a very high number of froghopper fossils, about 1200, suggest that froghoppers were abundant in their eco-systems. As observed in some 200 specimens with well-preserved structures of male and female genitalia, the genitalia of females and males were symmetrical in *A. perpetua* sp. nov., as shown by representative three male and four female paratypes in Figs. 3A–I, 4A–G. A single mating fossil from such a high number of specimens clearly underscores the rarity of fossils preserving behaviors.

As already argued by Alexander[26] and supported by Huber et al.[21], all evidence suggests that a female-above position is plesiomorphic for Neoptera. It is reported that mating position of extant Cicadomorpha is side-by-side, but the tips of male and female abdomens are connected the same as in the female-above position[20], [21], [27], [28]. Twisting of the abdomen or genitalia is involved in the side-by-side and the belly-to-belly positions. The fossil pair of copulating *A. perpetua* sp. nov. show belly to belly (or side by side) position, which is consistent with the mating position of extant froghoppers (Fig. 6A). The fossil also shows that the male abdominal segments 8 to 9 disarticulated from segment 7, suggesting that during mating, the male's abdomen was twisted and flexed after the 8^th^ segment, as depicted in a 3-D reconstruction Fig. 1B.

## Conclusions

In summary, our finding of the hitherto earliest record of copulating froghoppers, consistent with those of extant froghoppers, indicate froghoppers' genitalic symmetry and mating position have remained static for 165 million years. The evidence also confirms that symmetric genitalia are plesiomorphic for the taxon and by the Middle Jurassic, froghoppers have adopted the belly-to-belly (or side-by-side) position, which was proposed by Huber et al.[21] as the next step in the process of position changes from the basal female-above.
