# Correction: Forever Love: The Hitherto Earliest Record of Copulating Insects from the Middle Jurassic of China

**DOI:** 10.1371/annotation/54a6126f-eed2-456e-ba80-5a1fd2d78e8e

**Published:** 2014-01-06

**Authors:** Shu Li, Chungkun Shih, Chen Wang, Hong Pang, Dong Ren

In addition to institutions numbers 1 and 2, the first author, Shu Li, is also affiliated with institution number 3, State Key Laboratory of Biocontrol and Institute of Entomology, Sun Yat-sen University, Guangzhou, P.R. China. 

